# Using Social Marketing to Reduce Salt Intake in Iran

**DOI:** 10.3389/fpubh.2020.00207

**Published:** 2020-06-05

**Authors:** Mehdi Layeghiasl, Janmohamad Malekzadeh, Mohsen Shams, Mostafa Maleki

**Affiliations:** ^1^Department of Health Education and Promotion, Shiraz University of Medical Sciences, Shiraz, Iran; ^2^Department of Nutrition, Yasuj University of Medical Sciences, Yasuj, Iran; ^3^Social Determinants of Health Research Center, Yasuj University of Medical Sciences, Yasuj, Iran; ^4^Department of Health Education and Promotion, School of Health, Yasuj University of Medical Sciences, Yasuj, Iran; ^5^Iranian Social Marketing Association, Yasuj University of Medical Sciences, Yasuj, Iran; ^6^Department of Health Education and Promotion, School of Public Health, Tehran University of Medical Sciences, Tehran, Iran

**Keywords:** salt intake, salt reduction, formative research, social marketing, intervention

## Abstract

**Objectives:** WHO has recommended that the average salt intake must be <5 grams per day. However, people consume salt much more in many countries. In this study, we design and implement an intervention based on social marketing model to reduce salt consumption in Yasuj, Iran.

**Materials and Methods:** This study employed a quasi-experimental pretest-posttest with control group design which consisted of a formative research (qualitative-quantitative) and an interventional phase. To collect the qualitative data, six focus group discussions by participating of 66 people were established. The qualitative data were analyzed manually using directed content analysis. In quantitative study, 166 people aged 25–50 years completed a KAP questionnaire, and their average salt intake was determined through measuring sodium in their urine sample. By analyzing the data, marketing mix components were determined for designing an intervention. An educational package (including posters for installing in the kitchen, pamphlets, phone counseling, four educational classes, and brief interventions done by physicians and other health personnel) focused on reducing salt intake and using alternatives was developed. For one month, program was implemented for intervention group. Two months later, KAP survey and measuring the urine sodium were repeated for intervention and control groups. The data was compared for two groups, before and after the intervention by using independent t-test, paired t-tests and repeated measures ANOVA.

**Results:** The qualitative findings showed that most participants agreed that the salt intake was high in Iran. Most of them recommended home-based and family-driven strategies to reduce salt intake, offered using healthier alternatives for salt, and recognized physicians and health care providers in healthcare facilities as the most important to encourage people to reduce salt intake. After the intervention, the mean and standard deviation of KAP were improved significantly in intervention group. The mean salt intake decreased significantly by 3.01 ± 2.38 in the intervention group and repeated measures ANOVA showed significant change over time (*P* < 0.001) and a significant difference between two groups (*P* = 0.04)_._ Also, the interaction between time and group was significant (*P* = 0.001).

**Conclusion:** The mean salt intake among the study population was approximately three times more than the level recommended by the WHO. The social marketing-based intervention succeeded in reducing the salt intake of the study subjects by ~3 grams on average.

## Introduction

Cardiovascular diseases (CVDs) are the leading cause of death in the world. High blood pressure is the main modifiable biological risk factor of such conditions, and a high-salt diet is the most important risk factor for hypertension. Besides, excessive salt intake is directly associated with kidney disease, increased risk of obesity and osteoporosis, kidney stone formation and gastric cancer (1, 2). The World Health Organization (WHO) recommends that reducing salt intake should be considered as one of the three priorities to tackle the crisis of non-communicable diseases (3). To this end, the WHO has set a 30% reduction in the mean salt intake by 2025 in its global action plan for the control and prevention of non-communicable diseases (4).

On the other hand, the WHO has recommended that every adult should receive less than 5 grams of salt per day, provided the salt is iodized (5). However, studies show that the mean salt intake in most countries around the world is about 9 to 12 g/day, in many Asian countries it is more than 12 g/day (6). Surveys by the Canadian Public Health Association show that sodium intake in over 85% of men and 60% of women aged 19–70 years is over-recommended, leading to health risks (7). The mean salt intake among Iranians is 9.52 g/day (8).

Therefore, salt intake exceeding the recommended limit seems to be a global problem, which threatens the health of many people. Different countries have developed national guidelines for diet and salt intake. In the United States, it is recommended that the salt intake of each adult should be less than 6 g/day (9). Canada has targeted a 5.75 g/day salt reduction plan (10). The United Kingdom has planned interventions to reduce salt intake to 3 g/day by 2025 (3).

Studies in 23 low-income and middle-income countries have shown that a 15% reduction in the mean salt intake over 10 years could prevent 8.5 million CVD deaths, while a 20% reduction in smoking could only prevent 3.1 million CVD deaths (11). The annual cost of salt reduction strategies in Australia was estimated at up to $15 million, while the national blood pressure control program was estimated at more than $1 billion annually (12). Thus, reducing salt intake of the population is one of the most effective, cost-effective and easiest ways to reduce the risk of CVD and the cost of public healthcare, and this can significantly improve the general health status (2). Accordingly, reducing salt intake among people can be necessarily achieved by using new approaches, designing and implementing high-effectiveness interventions.

There are appropriate and effective strategies to reduce salt intake using audience-based planning, such as social marketing because of the focus of this model on audience views and the causes of the problem (13). The social marketing seeks to voluntarily encourage new behavior or modify existing behaviors by increasing perceived benefits and reducing perceived barriers (14). The social marketing conveys desirable ideas and behaviors to the target audience by focusing on the views, desires, and needs of the audience (15). The effectiveness of social marketing has been evident in many interventions, including increased physical activity, diet modification, increased consumption of fruits and vegetables, reduced consumption of alcoholic drinks, and cancer prevention (16–19).

Given the necessity of reducing salt intake among the Iranian population and adopting new approaches, we decided to design and implement a social marketing intervention to evaluate its effect on reducing the salt intake. We hypothesized that if the study group was exposed to a tailored social marketing intervention, their salt intake would be significantly reduced, while the salt intake would not change in the group that did not receive the intervention.

## Materials and Methods

### Social Marketing Assessment and Response Tool (SMART) Model

The model used in this study was SMART, whose phases are preliminary planning; audience, channel, and market analysis; educational materials development and pretesting; implementation; and evaluation. The preliminary planning phase was determined by identifying and detecting the problem and reviewing the resources available to the research team. The social marketing programs are broadly based on conducting formative research to design the interventions to ensure appropriate responses to the demands and needs of the target audience (19, 20). The formative research includes audience analysis, market analysis, and channel analysis, conducted in the present study in the form of a qualitative study (holding focus group discussions) and a quantitative survey (examining salt intake-related knowledge, attitude, and practice (KAP) as well as averaging daily salt intake in the participants). The characteristics of the target group and their views and comments on salt intake, elements of behavior marketing, appropriate and effective communication channels and initial ideas for intervention were identified during this formative study.

### Study Design and Population

This study employed a quasi-experimental pretest-posttest with control group design. The study consisted of exploratory phase (qualitative study, survey study and measuring daily mean of salt and sodium intake), implementation and evaluation phases (flowchart 1). The qualitative research and the related methodology and findings were published in a previous article (21). Therefore, this article reports only the quantitative survey and the intervention results.

The participants were selected from the population aged 25–50 years in Yasuj, Iran. Exclusion criteria were specific diets due to cardiovascular diseases, hypertension, and diabetes, failure to complete the questionnaire, refusal to give a urine sample, and unwillingness to continue to participate in the study for any reason. Inclusion criteria were a willingness to participate in the study and sign informed written consent. The study was approved by the University Ethics Committee, with the code of ethics of IR.YUMS.REC.1394.146.

### Exploratory Phase

#### Sample Size of Quantitative Survey

The purpose of the quantitative survey was to determine the mean salt intake by the households and to assess their (KAP) toward the salt intake. This part of the study had a cross-sectional design and therefore the sample size was determined using the formula proposed in the previous cross-sectional studies. The sample size was determined with a 95% confidence interval and an estimated error of the mean salt intake of 1 gr. In addition, we regarded the mean and standard deviation of salt intake (10 ± 5) reported in a study by Nazeri et al. (22). According to cluster sampling in different areas of the city, the research team considered the effect size of 1.5 in the study, and the dropout was estimated to be 10%. Accordingly, the final sample size was computed to be 166.

#### Sampling and Implementation Method in the Quantitative Survey

Multistage sampling as the probability sampling technique was used in this part of the study. Thus, the areas covered by each of the five healthcare centers in Yasuj were considered as one cluster and the sample size of each cluster was determined by proportionality. The interviewer referred to the cluster head, which was the first residential unit on the right of the street/alley under study, and completed the questionnaire for eligible individuals after obtaining written informed consent. Then, the interviewer left 10 residential units to complete the next questionnaire and continued this process until the cluster's share of sample size was completed. In the absence of the targeted study sample, the interviewer referred to the next housing unit, and this process continued until the contribution of that cluster was completed. The questionnaires were completed self-administered manner, except for justifiable reasons, such as inadequate literacy or inadequate dialect, requiring the interviewer to read the items.

Another part of the quantitative survey was to measure the mean salt intake of people in Yasuj before the intervention. To this end, the same people who completed the questionnaire were invited to their health centers for urine sampling. After obtaining written informed consent, the urine samples were collected daily in the morning and transferred to the laboratory. Urine sodium and creatinine levels were measured; based on the Kawasaki formula (23), the urine salt of each individual was calculated and finally, the mean salt level was determined for the study group.

#### Quantitative Survey Tool

The research team developed a questionnaire measuring the KAP toward salt intake with the participation of health education, nutrition and statistics specialists, and then calculating validity and reliability. Content validity index (CVI) and content validity ratio (CVR) were used to determine content validity. To calculate the CVR, the experts were asked to classify each item on a three-point Likert scale of “essential,” “useful but not essential,” and “not essential.” As 9 experts evaluated the items, items with a CVR of less than 0.78 were omitted. In calculating the CVI, it was also done by the experts to determine the “relevance,” “clarity,” and “simplicity” of each statement based on the Likert spectrum. The questions with a CVI of <0.79 were omitted. Finally, the CVI and the CVR were 0.89 and 0.93, respectively.

The reliability of the questionnaire was also assessed by Cronbach's alpha coefficient and autocorrelation coefficient. Thus, the questionnaire was given to 20 people in Yasuj. After one month, the questionnaire was again given to the same 20 persons. After inserting the data into SPSS version 2.3 software, the Cronbach's alpha was 0.97 in the knowledge questions, 0.98 in the attitude questions, and 0.97 in the practice questions. The questionnaire was used after confirming its validity and reliability, consisting of 3 demographic questions, 6 awareness questions, 10 attitude questions based on four-point Likert scale (strongly agree, neutral, disagree, and strongly disagree) and 5 practice questions based on four-point Likert scale (always, very often, sometimes, and never).

After performing the exploratory phase and analyzing qualitative and quantitative data, the behavioral intervention was designed, and conditions and facilities were provided for implementation.

### Intervention Phase, Implementation, and Evaluation of Intervention

With a type I error of 5% (Z1-α/2 =1.96) and a test power of 80% (Z = 0.845) considering the effect of the education program at 3 g reduction in the salt intake, the required sample size in the intervention implementation phase was 93 (for both the intervention and the control groups). With a 10% dropout, the final sample size was 103. It was supposed to random allocation was done according to the recommended level of salt intake (mean salt intake of 5 gr/day), in the other word the participants with the mean salt intake of 5 gr/day or more participated in the implementation phase. But, after the quantitative survey and the calculation of the mean salt intake, it was found that 100 % of the participants consumed the salt higher than the recommended level. Therefore, the research team decided to include all participants in the quantitative survey (*n* = 166) into the intervention implementation phase. In the other words, 63 participants allocated to the intervention group and 63 participant allocated to the control group randomly.

The intervention group received the intervention package for one month. The package consisted of posters for installing in the kitchen, pamphlets, free phone counseling, four educational classes, and brief interventions done by physicians and other health personnel. An educational package focused on reducing salt intake and using alternatives. The control group received routine interventions in healthcare centers. Based on the decision of the research team, the first session was held with the intervention group as face-to-face with the presence of the research team, who introduced the model and designed media, explained completely the project's implementation and duration, and invited them to continue the collaboration during the intervention. Two months after the intervention, KAP survey and measuring the urine sodium were repeated for the intervention and control groups.

### Social Marketing Mix

By analyzing collected data from the exploratory phase, practical insights were obtained. These insights guided the research team to develop an effective intervention tailored to the characteristics of the audience. In addition, the communication channels of the Yasuj people were identified and prioritized to convey program messages to them. The marketing components are as below:

Product: Behavior of salt intake reduction and use of salt substitutes.

Price: The training and intervention products were given to the target group for free with the least amount of time and at home easily to reduce costs and encourage them. It also sought to preserve the taste of the food by encouraging the use of salt substitutes.

Place: Training and interventions received at home and in health centers.

Promotion: Including logo design and use throughout the intervention period with the collaboration of physicians and healthcare providers in health centers, holding four training sessions with physicians and healthcare providers, and installing posters in the kitchen with daily and continuous reminder and making a decision with all family members, and performing brief intervention and telephone counseling.

Data collection after the intervention:

To assess the intervention effects, the KAP survey was conducted via the door-to-door referral by the trained interviewer two months after the intervention. In addition, the urine samples were obtained and sent to the laboratory for sodium and creatinine measurements from the same people who completed the questionnaire. The written-informed consent was read and signed by the participants before the data collection and urine sampling.

### Statistical Analysis

Collected data were analyzed using SPSS version 23. The normality of the data distribution was approved using plotting the histogram and calculating skewness and Kolmogorov-Smirnov test. Descriptive statistics were used to describe the demographic characteristics. Independent t-test was used to obtained the differences among participants in term of sex, and One-way ANOVA was conducted to check the significant differences among participants in term of age, educational level, KAP prior to the intervention implementation. Paired *t*-test and independent t-test were applied to compare the score of KAP between the intervention and control group before and after the intervention implementation. The repeated measures ANOVA was used to compare changes in outcome (salt and sodium intake) across time. We also examined the effect of time, group, and time–group interactions by using the repeated measures ANOVA.

## Results

### Quantitative Survey

The sample size of the quantitative survey was 166. Fifty percent of them were men and the other were women. The mean age and standard deviation of the participants were 36.4 ± 7.6. Table 1 shows all the demographic characteristics of the subjects studied.

**Table 1:** Demographic description of the participants in the survey study.

**Demographic variables**		**Number (Percentage)**
Sex	Male	83 (50)
	Female	83 (50)
Education level	Illiterate	14 (8.4)
	Primary school	18 (10.8)
	Secondary school	27 (16.3)
	High school	62 (37.3)
	University degree	45 (27.2)
Age	25–29 years old	38 (22.9)
	30–34 years old	38 (22.9)
	35–39 years old	34 (20.5)
	40–45 years old	25 (15)
	45–50 years old	31 (18.7)

Totally, the mean salt intake was 14.34 ± 3.56 g/day at baseline and 11.33 ± 2.11 two months after the intervention.

The salt intake ranged from 7 to 21 g/day. As can be seen in Table 2, the mean daily salt intake among all participants was higher than the recommended level of the WHO. Even the lowest daily salt intake was higher than the recommended level of the WHO.

**Table 2:** Comparison of mean and standard deviation of salt intake among participants based on demographic variables and KAP survey.

**Demographic variables**		**Mean ± S.D**	***P*-value**
Sex	Male	13.58 **±** 3.36	^*^0.12
	Female	14.42 **±** 3.74	
Education level	Illiterate	15.14 **±** 4.45	^**^0.11
	Primary school	15.50 **±** 3.33	
	Secondary school	13.74 **±** 3.82	
	High school	14.03 **±** 3.18	
	University degree	13.16 **±** 3.41	
Age	25–29 years old	13.84 **±** 3.82	^**^0.31
	30–34 years old	13.18 **±** 2.59	
	35–39 years old	14.82 **±** 2.88	
	40–44 years old	13.72 **±** 4.00	
	45–50 years old	14.52 **±** 4.25	
Knowledge	Poor	13.30 **±** 3.20	^**^0.18
	Moderate	14.08 **±** 3.53	
	Good	14.83 **±** 3.87	
Attitude	Poor	15.75 **±** 3.53	^**^0.2
	Moderate	14.13 **±** 3.57	
	Good	13.50 **±** 3.40	
Practice	Poor	14.31 **±** 3.77	^**^0.68
	Moderate	14.02 **±** 3.60	
	Good	13.09 **±** 2.54	

* *Obtained from Independent T-test*.

** *Obtained from ANOVA*.

The mean salt intake was 15.14 g/day in illiterate individuals, which was 1.14 g/day higher than the total mean. Those with the educational level of primary school had the highest salt intake and those with academic education had the lowest salt intake. Among the age groups, people aged 35–39 years had the highest salt intake and those in the age group 30–34 years had the lowest salt intake. However, there was no significant difference between the mean salt intake in terms of age, gender, and educational level. Besides, the mean daily salt intake was higher in participants who had poor KAP. Overall, based on ANOVA there was no significant difference in the mean salt intake distribution by knowledge, attitude, and practice (Table 2).

### Findings From the Intervention Evaluation

According to the paired t-test, the mean score of knowledge after intervention in the intervention group was significantly increased (mean ± standard deviation of change = 2.58 ± 1.3, *P* = 0.001), while the mean score of knowledge after intervention in the comparison group was not significant (mean ± standard deviation= 0.012 ± 0.33). In addition, after the intervention, the mean score of attitude (mean ± standard deviation of change = 1.68 ± 4.03, *P* = 0.001) and practice (mean ± standard deviation of change = 3.37 ± 2.92, *P* = 0.001) also changed significantly among intervention group, but this value did not change significantly in the comparison group (Table 3).

**Table 3:** Comparison of mean and standard deviation of knowledge, attitude, and practice between intervention and comparison group before and after the intervention.

**Variables**	**Group**	**Before the intervention Mean ± S.D**	**After the intervention Mean ± S.D**	**Changes Mean ± S.D**	^*^P-value
Knowledge	Intervention	2.46 ± 1.1	5.04 ± 0.97	2.58 ± 1.3	0.0001
	Comparison	2.27 ± 1.15	2.25 ± 1.09	0.01 ± 0.33	0.74
	^**^Sig.	*P-*value = 0.27	*P-*value = 0.0001	*P-*value < 0.0001	
Attitude	Intervention	21.6 ± 3.1	23.3 ± 3.6	1.68 ± 4.03	0.0001
	Comparison	22.13 ± 2.7	22.01 ± 2.9	0.12 ± 1.01	0.28
	^**^Sig.	*P-*value = 0.28	*P-*value = 0.01	*P-*value < 0.0001	
Practice	Intervention	5.04 ± 1.8	8.42 ± 2.86	3.37 ± 2.92	0.0001
	Comparison	5.46 ± 2.25	5.43 ± 2.19	0.03 ± 0.88	0.71
	^**^Sig.	*P-*value = 0.19	*P-*value = 0.0001	*P-*value < 0.0001	

* *Obtained from Paired T-Test*.

** *Obtained from Independent T-Test*.

The effect of an intervention on salt and sodium was shown in Table 4. According to the repeated measures ANOVA, both the salt and sodium intake resulted in a significant reduction. The salt and sodium level reduction was significant two months after the intervention (*P*_time_ = 0.001). In addition, there was a significant difference between two groups (*P*_group_ for the salt level = 0.04, and *P*_group_ for sodium level = 0.001). Also, the interaction between time and group was significant (*P*_time × *group*_ = 0.001).

**Table 4:** Comparison of mean and standard deviation of sodium and salt between intervention and comparison group before and after the intervention.

**Variables**	**Group**	**Before the intervention Mean ± S.D**	**After the intervention Mean ± S.D**	**Changes Mean ± S.D**	^*^P-value
Sodium	Intervention	155.05 ± 58.21	115.69 ± 48.11	39.36 ± 37.96	0.0001
	Comparison	162.28 ± 50.25	166.02 ± 51.15	3.74 ± 23.19	0.14
	^**^Sig.	*P-*value = 0.39	*P-*value = 0.0001	*P-*value < 0.0001	
Sodium chloride	Intervention	14.34 ± 3.65	11.33 ± 2.11	3.01 ± 2.38	0.0001
	Comparison	13.66 ± 3.38	13.84 ± 3.22	0.18 ± 0.84	0.05
	^**^Sig.	*P-*value = 0.21	*P-*value = 0.0001	*P-*value < 0.0001	

* *Obtained from Paired T-Test*.

** *Obtained from Independent T-Test*.

## Discussion

The current study aimed to investigate the effect of social marketing intervention on reducing the salt intake among the subjects aged 25–50 years in Yasuj. The qualitative study and its' results have been already reported in a separate article, but the findings of this part of the study are discussed here. Participants rated their salt intake very low or stated that they were unaware of their salt intake, which could be one of the most important causes of their excess salt intake.

Similarly, some other studies found that people underestimate their salt intake and are unaware of the main sources of salt intake (24–26). This can be one of the main reasons for getting too much salt. Importance of food taste and taste sensitivity were other barriers to reducing salt intake in the present study, with most participants finding it to be a major contributor to high salt intake. Family members' preferences, especially the spouse, about the food taste were another barrier to the salt reduction, which the interviewees repeatedly stated. similar to our findings, results of a qualitative study by Ponce-Lucero revealed that the change in the flavor of the food was stated as the most difficult challenge to salt reduction (27). The interviewees in this study also suggested holding courses as one of their suggestions. Many participants offered using alternatives to reduce salt intake (21).

The findings of the quantitative survey showed that the mean daily salt intake among the participants was 14 ± 3.52 g/day. The first national report of salt intake in Iran demonstrated that Iranians receive an average of 9.52 g of salt per day (8). Studies in different provinces of Iran showed that the mean daily salt intake is higher than the recommended amount of the WHO. For example, people in Yazd receive about 10.9 g/day (28), the trend of mean salt intake in Isfahan from 1998 to 2013 revealed that it was increased from 9.5, 9.7, 9.6, and 10.2 g/day, respectively (29). Azizi et al. reported that the mean salt intake in housewives aged over 20 years in Rasht and Sari were 7.50 ± 4.72 and 7.75 ± 4.02, respectively (30). It can be concluded that most men salt intake among Iranians' people is very high and an effective-community based intervention is essential to reduction of it.

The mean salt intake is about 9–12 g/day in most countries around the world (6) and it is estimated that the mean salt intake is 10.06 g/day worldwide (31). The mean salt intake is more than 12 g/day in many Asian countries (6). For example, a study found that the mean salt intake was 12.4 ± 3.1 g/day in Japanese women (31). Surveys by the Canadian Public Health Association revealed that the sodium intake in over 85% of men and 60% of women aged 19-70 years is over-recommended, leading to health risks (7). An updating evidence on global estimates of salt intake showed that Mean daily salt intake ranged from 6.75 g/d in Barbados to 10.66 g/d in Portugal (32).

In the present study, there was no significant difference between mean salt intake and demographic variables of age, gender, and educational level and KAP toward salt intake. In line with the results of the present study, in study of by Hasenegger, no significant differences in salt intake were observed for affluence, educational level (33). In another similar study in Iran, there was no significant relationship between the salt intake behaviors and the demographic variables (age, occupation, spouse occupation, educational level of a spouse, and household dimension) (34). Unlike our findings, Nasredine and et al. stated that there was a significant correlation between age and gender with salt-related behaviors, such that adopting desirable behaviors was more common among women aged over 51 years (35). Therefore, it seems that the level of salt intake by the studied subjects is more influenced by other factors than the ones were studied. The taste and food culture of people may be more decisive factors.

The findings of the present study indicate that the majority of participants have low knowledge of the complications of high salt intake. Similar other studies also revealed that the general level of knowledge of people about salt is low (36, 37). Studies in Iran have also shown that people have insufficient information about the recommended daily intake of salt (28, 37). In this study, people with poor attitude had the highest daily salt intake. Mazlumi also confirms this, and the attitude was the most important determinant of salt intake (34).

The results of the intervention evaluation showed that the salt intake by 3.01 ± 2.38 g/day two months after the intervention, which is consistent with the findings of other studies. In a study of Moini targeting women at risk of hypertension referred to health centers in the city, the mean salt intake in the intervention group was decreased significantly by 1.54 g/day after two months of intervention (23). In a study by Juan Chen, who sought to reduce salt intake through a small spoon of salt and health education based on the constructs of the health belief model, it was found that the urine sodium decreased 1.42 g/day in the intervention group after 6 months of intervention (38). Other studies also report that the implementation of intervention programs with the aid of behavioral change theories resulted in a significant reduction in salt intake (39–41). Along with our results, an intervention by Pillay in Fiji was succeed to reduce the mean salt intake by 1.4 g/day (42). A study based on theory of planned behavior could reduce the mean salt intake by 4.73 g/day in Yazd, Iran (43).

One of the highlights in the present study was salt intake higher than the recommended level in 100% of participants, and there is a need for further investigation of the causative factors. This finding is line with the study which assessed the trend of mean salt intake in Iranian adults. In this study, it was found that more than 95% of our participants consumed higher than WHO recommendation (29). It may be argued that the cultural status of the people of Yasuj and their taste and interest in salty taste are some of the possible reasons. This could also be an alarm for public health in the province, and if comprehensive and ongoing plans to reduce salt intake are not implemented, the public health in the province will be at potential risk.

One of the most effective ways to reduce salt intake is to organize social marketing campaigns and interventions to increase knowledge of the recipients and help them to choose healthy food. As the WHO also recommends, in addition to these interventions, food products from the food factories should be produced in a new formulation to reduce the salt levels used in food production gradually and continuously. Besides, policymakers and decision-makers must support an environmental change to provide healthier food with easier access and at an affordable cost (11). Argentina, Canada, and Chile have significantly reduced the salt intake of their people by using these strategies (44).

The present study also had some limitations. A 24-h urine collection is the gold standard for the measurement of salt intake, but we used a random urine collection method in the present study to estimate mean sodium and salt intake. Household economic status was one of the variables considered by the research team to purposefully classify the participants in group discussions, but the research team was forced to disregard this variable because of the participants' lack of clear responses to the issue, and the lack of accurate information on their economic status in the household file. Furthermore, Due to a very unavailable geographic distribution in rural areas, we decided to recruit the participants from urban residents.

According to the findings from the present study, it can be concluded that the mean salt intake among the study population was approximately three times more than the level recommended by the WHO and that the social marketing-based intervention succeeded in reducing the salt intake of the study subjects by ~3 grams on average.

## Data Availability Statement

The datasets generated for this study are available on request to the corresponding author.

## Ethics Statement

The studies involving human participants were reviewed and approved by Ethics Committee of the Vice-Chancellor's Office for Research at the Yasuj University of Medical Sciences. The patients/participants provided their written informed consent to participate in this study.

## Author Contributions

MS took responsibility for the integrity of study, conceived the statistical methodology, drafted the manuscript, conceived the study design and performed the final writing of the paper. JM conceived the statistical methodology. ML was the executive manager of the study, collected the data, helped writing the manuscript draft, MM conceived the study design and perform the final writing of the final version and followed the modification of the revisions. All authors read and approved the final version of the manuscript.

## Conflict of Interest

The authors declare that the research was conducted in the absence of any commercial or financial relationships that could be construed as a potential conflict of interest.
